# Arachidonic Acid Derivatives and Their Role in Peripheral Nerve Degeneration and Regeneration

**DOI:** 10.1100/2012/168953

**Published:** 2012-09-10

**Authors:** Carlos Rodrigo Camara-Lemarroy, Emmanuel Irineo Gonzalez-Moreno, Francisco Javier Guzman-de la Garza, Nancy Esthela Fernandez-Garza

**Affiliations:** ^1^Departamento de Medicina Interna, Hospital Universitario “José Eleuterio González”, Universidad Autónoma de Nuevo León, School of Medicine, Avenida Francisco I. Madero y Dr. Eduardo Aguirre Pequeño S/No, Colonia Mitras Centro, 64460 Monterrey, Nuevo León, Mexico; ^2^Departamento de Fisiologia, Hospital Universitario “José Eleuterio González”, Universidad Autónoma de Nuevo León, School of Medicine, Avenida Francisco I. Madero y Dr. Eduardo Aguirre Pequeño S/No, Colonia Mitras Centro, 64460 Monterrey, Nuevo León, Mexico

## Abstract

After peripheral nerve injury, a process of axonal degradation, debris clearance, and subsequent regeneration is initiated by complex local signaling, called Wallerian degeneration (WD). This process is in part mediated by neuroglia as well as infiltrating inflammatory cells and regulated by inflammatory mediators such as cytokines, chemokines, and the activation of transcription factors also related to the inflammatory response. Part of this neuroimmune signaling is mediated by the innate immune system, including arachidonic acid (AA) derivatives such as prostaglandins and leukotrienes. The enzymes responsible for their production, cyclooxygenases and lipooxygenases, also participate in nerve degeneration and regeneration. The interactions between signals for nerve regeneration and neuroinflammation go all the way down to the molecular level. In this paper, we discuss the role that AA derivatives might play during WD and nerve regeneration, and the therapeutic possibilities that arise.

## 1. Introduction

Nerve injury can occur at any point along the length of a peripheral nerve as it courses from the root through the plexus and then to the target organ. There are a number of mechanisms whereby peripheral nerves may be directly traumatized, including compression, traction, drug injection, and laceration, toxins, ischemia, infection, and physical agents. The principal target of peripheral nerve injury is the axon. Injury may also affect specialized neuronal sheath cells called Schwann cells (SCs), which are intimately associated with all peripheral nerve axons. Irrespective of cause, there is a limited range of responses to peripheral nerve injury of which the most important is Wallerian degeneration (WD).

WD is a sequential pattern of axonal degeneration, myelin degradation, and supporting glial cell proliferation lasting 24–48 h. During this complex process, various events take place, including blood-nerve barrier dysfunction, endoneural space reorganization [1], and most importantly for our purposes, the induction of an intense inflammatory response, constituted by inflammatory mediator release and production [2]. Axonal degeneration initiates this response, activating SC and macrophages, that prolipherate and activate, clearing myelin debris and producing cytokines that perpetuate an inflammatory state. Axonal regeneration is then regulated by the interactions between all the involved cell types and by cytokines, chemokines, growth factors, and other inflammatory mediators [2]. All these events culminate in the promotion of an environment suitable for subsequent regeneration, repair, and axon regrowth.

Arachidonic acid (AA) and its metabolites are known to modulate neuronal function and survival. There is also evidence that AA derivatives, such as prostaglandins (PG), leukotrienes, and the enzymes involved in their production, such as cyclooxygenases (COX), lipooxygenases (LOX), among others, are centrally involved in WD and in axonal regeneration [2]. In this paper we will discuss the available evidence that sheds light in this issue.

## 2. Phospholipases and AA

Phospholipases (PL) are ubiquitous in mammalian cells and serve to cleave free fatty acids from cell membrane phospholipids. AA is one such fatty acid, and itself a precursor for eicosanoids. PLs are known to be upregulated in neurons weeks after crush injury to peripheral nerves, indicating increased protein synthesis involved in regeneration [3]. PLA has been hypothesized to participate in neuronal membrane disruption after injury, via lypolisis, DNA fragmentation, and lipid peroxidation, through a calcium-dependent mechanism [4, 5].

PLA is expressed in the nerve crush site as well as in resident and infiltrating macrophages, suggesting a role for PLA in myelin breakdown, a vital process during WD [6]. PLAD1 immunoreactivity is also increased in SCs and macrophages in sciatic nerves, using a rat model of experimental neuritis [7]. Recent evidence has established that PLA2 initiates the breakdown of compact myelin through macrophage interactions and participates in chemokine and cytokine expression after nerve injury [8]. PLs are also known to participate in the molecular signaling of SC morphology and proliferation [9], and immortalized SCs show increased PLC activity [10]. PLC alpha shows a similar pattern of increased expression during the first days after axonal injury, while PLC beta-1 expression is reduced in the same setting [11], pointing to different functions and dynamics of PLs. In keeping with these results, knockout and pharmacological inhibition studies have established specific roles for different PLA2 families during WD. The calcium-independent group VIA participates in the early stages of myelin breakdown, while the calcium-dependent group IVA participates in myelin clearance and phagocytosis by macrophages [12]. However, the accumulated evidence leaves little doubt of the participation of PL during nerve degeneration/regeneration.

The role of PLA2 during axonal regeneration was further clarified in studies showing that PLA2 inhibitors diminish neuron outgrowth after axonal injury, and that PLA2 activators seem to promote it [13]. Similar findings were described in brain noradrenergic injured neurons, where PLA2 activators could induce axonal regeneration [14]. Coupled with evidence of PLA2 expression in growth cones, this evidence points toward a local role for PLA in nerve regeneration. PLA also participates during degeneration. Mutant mice with impaired WD do not show PLA expression in injured nerves, while mutant mice with PLA deficiencies show diminished myelin and axonal degradation and phagocytosis [15]. Additionally, whereas increased expression of PLA is common in peripheral nerve after injury, the same is not evident after injury to the optic nerve, which correlates to slow WD in the central nervous system (CNS).

AA itself has been found to posses trophic and toxic effects both in hippocampal neurons and in chick motoneurons *in vitro*, possibly trough a LOX pathway [16, 17]. AA can also participate in neurite growth trough a calcium-dependent mechanism [18].

There is another potential mechanism by which PL and AA contribute to the pathophysiology of nerve degeneration. The oxidative metabolism of AA is known to result in the production of free radicals and in lipid peroxidation. These processes are partly responsible for the initial local damage induced by acute injury in nerve tissue, due to the rich lipid content of myelin sheats. The full implication of this association remains to be elucidated.

## 3. COX 

COXs catalyze the conversion of AA to PGs and thromboxanes, which trigger as autocrine and paracrine chemical messengers in many physiological and pathophysiological responses [19]. The COXs exist in two isoforms, a constitutive form (COX-1) and an inducible form (COX-2), and a COX-1 splice variant termed as COX-3 has been reported [20]. COX-1 and -2 share the same substrates, produce the same products, and catalyze the same reaction using identical catalytic mechanisms [21, 22]. The COX-1 enzyme is expressed in most tissues and is responsible for maintaining homeostasis (gastric and renal integrity) and normal production of PGs. COX-2 is predominantly found in brain, kidney, and endothelial cells and is significantly upregulated as part of various acute and chronic inflammatory conditions. COX-2 expression can be induced in response to growth factors, cytokines, and proinflammatory stimuli. Moreover, COX-2 has been implicated in the regulation of the inflammatory response that occurs during WD, both directly and by way of their products, eicosanoids.

After nerve injury, COX-2 expression is locally upregulated, but this phenomenon has been mainly linked to the pathogenesis of neuropathic pain [23, 24], although COX-1 could also participate in tissue remodeling during WD in the spinal cord [25]. Both COX-1 and COX-2 expression is increased in macrophages and SCs in animal models of inflammatory demyelinating diseases [7]. The administration of COX inhibitors obstruct myelin debris signals that negatively affect axonal regeneration after injury, resulting in increased axonal regeneration *in vitro*, and COX inhibitors can also affect SC and macrophage phagocytic activity [26, 27]. In a recent paper, we reported accelerated functional recovery after sciatic nerve crush in rats treated with celecoxib, a selective COX-2 inhibitor, thereby directly implicating COX-2 in nerve regeneration [28].

## 4. Eicosanoids

After nerve injury, increased COX expression induces the production of PG in nerve terminals and nonneural cells in the surrounding areas, a process that is known to induce hyperalgesia and neuropathic pain [23, 29]. PGs are produced in important quantities, and for prolonged time periods, both directly by injured nerves, as well as by macrophages in response to soluble factors produced by injured nerves [30]. Prostanoid receptors, effectors of biological actions of PG, have been shown to be expressed in SCs, and to be able to modulate SC function *in vivo* [31]. Additionally, studies have demonstrated that PGE2 is able to modulate microglial migration and function [32]. Prostanoids have also been shown to interact with nerve growth factor in the regulation of inflammatory responses and degeneration after tissue injury [33]. Coupled with the pivotal roles that microglia play during WD and regeneration, a role for PGs in nerve degeneration and regeneration after injury is beginning to be recognized.

PG are vasoactive molecules, and their contribution to blood-flow homeostasis and inflammation during nerve injury could be important. Alprostadil, a PGE1 analogue, diminishes peripheral nerve ischemia-reperfusion injury, probably through such a mechanism [34]. Other mechanisms are involved during nerve repair, however. After nerve crush injury, alprostadil treatment results not only in reduced injury, but also in increased repair rates and in the upregulation of vascular endothelial growth factor, itself known to be neuroprotective [35]. PGD2, a potent inflammatory mediator, shows increased expression in macrophages after spinal cord injury, and both PGD2 inhibition and PGD2 knockout result in improved locomotor function [36]. 

The induction of neuronal apoptosis after nerve injury is an established phenomenon that contributes to the physiopathology of nerve degeneration. The administration of PGE1 inhibits neuronal apoptosis in the spinal cord after sciatic nerve constriction injury, independently of changes in local blood flow [37]. This suggests a mechanism not solely dependent on vasoactive properties of PG. Further studies also showed that not only does PGE1 reduce apoptosis but also improves neuronal regeneration after sciatic nerve crush injury [38]. Latanoprost, a PGF2 analogue, could also reduce retinal ganglion cell apoptosis after optic nerve crush [39, 40]. This evidence supports the idea that PGs are neuroprotective through the inhibition of apoptosis.

The mechanisms by which PGs modulate nerve regeneration seem to be quite complex. PGs are known to modulate the upregulation of heat-shock protein-70 expression, a protein response known to participate in the maintenance of neuron survival after nerve injury [41, 42]. After nerve injury, macrophages migrate into the area and initiate degenerative and regenerative processes. PGE2 has been demonstrated to modulate the production of interleukin-6 in invading macrophages after nerve injury, a cytokine known to participate both in nerve degeneration and regeneration [2, 43]. In the central nervous system, PGA1 is neuroprotective against injury through a variety of mechanisms, such as blockade of the proinflammatory transcription factor nuclear factor *κ*appa B (NF-*κ*B) and upregulation of peroxisome proliferator-activated receptor-gamma [44]. Considering the important role of NF-*κ*B in peripheral nerve degeneration and regeneration, this mechanism could be biologically relevant [2].

Much evidence exists to support the idea that beyond hyperalgesia and blood-flow regulation, PGs also contribute to the molecular and cellular process of nerve degeneration and regeneration. However, current knowledge is limited, and the exact mechanisms are just being uncovered.

## 5. LOX

LOX and its metabolites are known to participate in the physiopathology of many disorders in the central nervous system and in acute inflammatory disorders [45], but they also play a role in neurite growth, an essential step in axonal regeneration. Leukotrienes are produced in injured nerves and regulate neuropathic pain in a similar fashion as in the case of PGs [46], and SCs produce leukotrienes in response to inflammatory signaling [47]. Hepoxilin A3 (HxA3), is a 12-lipoxygenase metabolite of AA, found in the mammalian nervous system, and has been proposed to play a global role in calcium regulation [48]. Hepoxilin enhances neurotrophin-dependent neurite regeneration in cultured axotomized neurons, possibly through the modulation of intracellular calcium, which plays a crucial role in neurite outgrowth during development and regeneration, including gene expression, cytoskeleton assembly, and growth cone formation [49, 50]. Axonal injury also induces PLA activity through a calcium-dependent mechanism, and leukotrienes result as second messengers that control growth cone formation [51]. In fact, both leukotriene antagonists and PLA inhibitors result in delayed neurite outgrowth and function in cultured neurons [52]. Leukotriene B4 induces the differentiation of neural stem cells into neurons that actively produce neurite outgrowth [53]. Together these findings implicate LOX metabolites in the process of axonal regeneration.

## 6. AA-Associated Neuroinflammation and Regeneration Signaling at the Molecular Level

Neurons in the peripheral nervous system (PNS) respond to injury through gene expression in an appropriate regeneration-prone environment as well [54]. The mediators of these dynamics are transcription factors and their networks, that act as orchestrators of gene expression according to intrinsic nerve regeneration programs. This is thought to be the fundamental difference between CNS and PNS neurons in terms of regenerating capacity. Considering the complexity of these processes, it is no surprise that they interact and sometimes merge with the same signaling that mediates inflammation after PNS injury. 

Activating transcription factor 3 (ATF3) is a stress-inducible transcription factor known to be induced after injury in neurons, and a regulator of neuron growth in its constitutive from as well. COX inhibition is known to alter the expression of ATF3 in human colorectal carcinoma cells and thus inhibit growth [55]. Signal transducer and activator of transcription 3 (STAT3) is activated in response to growth factors, cytokines, and hormones that are known to play protective role after nerve injury and to mediate nerve regeneration [54]. COX-1 deficient mice show reduced neuroinflammatory and microglial responses to insults, through reduced activation of both Nf-*κ*B and STAT3 [56]. PGE2 is known to regulate the activation of STAT3, and PGE2 inhibition alters a signal required for dendritic cell survival and development, leading to apoptosis [57]. Survivin, a regulator of cell survival, is regulated by COX-2-generated PGE2 in part trough a STAT3 pathway [58]. PGE2 also mediates survival of nonneural cells, such as myocytes, through a STAT3 pathway [59]. 

The phosphoinositide 3-kinase (PI3K) and the mitogen-activated protein kinase (MAPK) pathways are activated in injured neurons, and act as intracellular cascades that modulate regeneration associated genes. Indomethacin treatment in tumor cells has been shown to decrease expression of PI3K [60]. Latanoprost, the synthetic derivative of PGF2a, is able to promote retinal ganglion cell survival and promote neurite outgrowth through a PGF receptor-mediated modulation of the PI3K pathway [61]. PI3K inhibition leads to enhanced protsnoids production in activated microglia, and additionally, PI3K can regulate the expression of COX-2 in microglia as a response to neuroinflammation [62]. MAPK pathways are involved in neuroinflammation in many ways, including the enhancement of sensory neuron interleukin-6 production [63] and the regulation of interleukin-1-mediated COX-2 expression [64]. Latanoprost also uses a MAPK pathway in order to rescue nuroglial cells from apoptosis [65]. 

Specificity protein-1 (Sp1) is a transcription machinery theorized to act as a general regulator of many of the nerve injury-inducible transcription factors, including some discussed above [54]. COX inhibitors are also known to alter Sp1 phosphorylation and lead to arrested cell growth in tumor cells [66], and PGE2 can stimulate cell growth through induction of Sp1 DNA-binding activity [67]. PGE2 enhances the phosphorylation, DNA binding, and transcriptional activity of Sp1, and it leads to enhanced neurotrophin production through a Sp1 pathway [68], suggesting a possible mechanism for PGE2-induced reinnervation. 

These finding illustrate the complex interactions between inflammatory and neuroregenerative signaling. Furthermore, these mechanisms could partly enlighten some of the ways AA derivatives might further regulate the regeneration-prone environment and suggest additional strategies in order to modulate neuroinflammation, and therefore, nerve survival after injury. 

## 7. Therapeutic Possibilities

**Figure 1:**
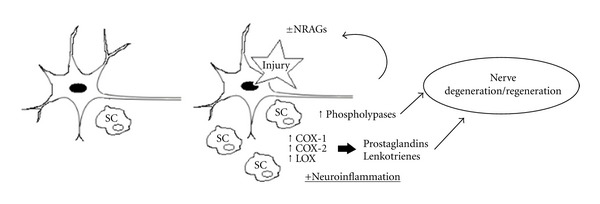
Proposed theoretical framework. After nerve injury, AA derivatives produced in neurons and microglia participate in nerve degeneration and regeneration, through local, remote, and molecular pathways. Injury promotes SC infiltration and activation as well as phospholipase, COX and LOX upregulation with the ensuing production of eicosanoids. These signals promote degeneration initially and later regulate the regenerative process. NRAGs are in turn heavily influenced by these signals. SC: Schwann cell; NRAGs: nerve regeneration associated genes; LOX: lipooxygenases; COX: cyclooxygenases.

The use of COX inhibitors for posttraumatic neuropathic pain is an established therapeutical intervention. However, given the association that exists between AA derivatives and nerve degeneration and regeneration, the therapeutic modulation of this pathway emerges as a novel strategy aimed at increased motor, sensory, and structural recovery after nerve injury. We summarize some of the potential mechanisms involved in Figure 1. Currently, COX inhibitors, LOX inhibitors, PG analogues, among other drugs, are approved for human use in clinical situations (Table 1). The development of phospholipase modulators and other drugs targeting AA derivatives is an active field of research, which will soon clarify the validity of this therapeutic strategy.

The dual function of AA derivatives, as promoters and inhibitors of nerve degeneration and recovery, will complicate the clinical application of these drugs. It is becoming evident that the dual role of neuroinflammation as both injurious and as a promoter of regeneration is a complex one, even at the molecular level. It is most likely that a combination approach will be fruitful, using compounds that optimize regeneration and others that diminish degeneration. The exact time of administration will also be important, since some of these molecules are expressed early after injury, while others begin to be produced in the late stages after regeneration. Additionally, although the evidence reviewed here concerns primarily peripheral nerve injury, most of the concepts could be applied both to spinal cord and CNS injury. In conclusion, the findings reviewed here point toward a new avenue in the pharmacological treatment of nerve injury, based on a pathophysiologically relevant paradigm (Table 2). The numerous compunds that exist today suggest that clinical studies are warranted.

## Figures and Tables

**Table 1:** Clinically approved modulators of arachidonic acid derivatives.

Drug class	COX inhibitors	Eicosanoid analogues	LOX inhibitors and leukotriene antagonists	Phospholipase inhibitors
Examples	NSAIDS Selective COX-2 inhibitors (celecoxib)	Misoprostol (PGE1) Latanoprost (F2*α*) Unoprostone (F2*α*) Travoprost (F2*α*) Iloprost (PGI2) Epoprostenol (PGI2)	Zileuton Zafirlukast Montelukast	Quinacrine Mepacrine Chloroquine

Clinical applications	Analgesic Anti-inflammatory	Pulmonary hypertension (iloprost, epoprostenol) Glaucoma (latanoprots, unoprostone)	Asthma, allergic rhinitis, urticaria	Antiprotozoal Antirheumatic Antimalarial

Routes of administration	Oral, parenteral	Inhaled, parenteral, topical	Oral	Oral

Notes			Zafirlukast and Montelukast are cysteinyl leukotriene receptor CysLT1 antagonists	

NSAID: nonsteroidal anti-inflammatory drugs; PGI: prostacyclin.

**Table 2:** Selected animal and human therapeutic studies.

Reference number	Model	Intervention	Outcome
[26]	Spinal cord injury in rats	Indomethacin and ibuprofen (COX inhibitors)	Increased functional recovery and axonal regeneration
[28]	Sciatic nerve crush in rats	Celecoxib (COX2 inhibitor)	Improved functional recovery
[38]	Sciatic nerve crush in diabetic rats	PGE1	Improved axonal regeneration
[37]	Sciatic nerve constriction injury in rats	PGE agonist	Decreased neuronal apoptosis
[35]	Sciatic nerve injury in rats	Alprostadil (PGE)	Decreased injury and improved functional recovery
[34]	Sciatic nerve ischemia-reperfusion injury in rats	Alprostadil	Decreased injury
[69]	Carpal tunnel syndrome. Human clinical trial	Tenoxicam (COX inhibitor)	No beneficial effects compared to placebo
